# The Impact on Audience Engagement of Coordinating a Public Health Campaign on Antimicrobial Resistance Through a Network of Health Content Creators: Longitudinal Observational Study

**DOI:** 10.2196/86587

**Published:** 2026-03-27

**Authors:** Fangyue Chen, Jack Cooper, Amish Acharya, Simon Dryden, Ara Darzi, Kate Grailey

**Affiliations:** 1 Fleming Initiative, Institute of Global Health Innovation, Imperial College London, QEQM Building, St Mary's Hospital, South Wharf Road, London, W2 1PE, United Kingdom, 44 7510888677

**Keywords:** health campaign, audience engagement, social media, antimicrobial resistance, health content creator

## Abstract

**Background:**

Antimicrobial resistance (AMR) is a significant global health threat. Several public health campaigns aimed to raise AMR awareness and inspire related behavioral changes have been delivered in a time-specific, coordinated manner, while others have placed less emphasis on campaign timing. Social media platforms can be leveraged as key vehicles for delivering public health campaigns, particularly by collaborating with health content creators who serve as influential messengers. Increasingly, organizations such as the World Health Organization and TikTok have created health content creator networks; however, the impact of such networks in public health campaigns, especially when delivered in a coordinated, time-specific manner, remains uncertain.

**Objective:**

This study aimed to investigate whether mobilizing an established health content creator network to create social media content on the topic of AMR, released in a coordinated, time-specific manner, can have an impact on audience engagement.

**Methods:**

We conducted a longitudinal observational study evaluating the effect of a coordinated social media campaign (“Pulse”) on YouTube, delivered by an established health content creator network during an international event on AMR. Members of the network prepared and coordinated the release of AMR-related videos. Engagement analytics were evaluated 6 months after release. The engagement with each campaign video was compared with that for a similar noncoordinated video and with the average engagement of the same creators.

**Results:**

Around the day of the Pulse campaign on September 26, 2024, 18 campaign videos were released across 14 YouTube channels. Compared with paired videos, Pulse videos were not associated with higher view counts (incidence rate ratio [IRR] 0.98, 95% CI 0.44-2.13; *P=*.95) or like counts (IRR 1.10, 95% CI 0.48-2.41; *P=*.81) but were associated with significantly higher comment counts (IRR 2.99, 95% CI 1.02-8.52; *P*=.03). When compared with the creators’ 12-month channel averages, campaign videos had a significantly higher comment count (IRR 15.5, 95% CI 5.5-24.0; *P*<.001) but no difference in view counts (IRR −82.0, 95% CI −190.3 to 58.5; *P*=.26) or like counts (IRR −0.50, 95% CI −6.3 to 10.5; *P*=.93).

**Conclusions:**

Coordinating health content creators to release AMR-related videos on YouTube coinciding with an international AMR event increased audience interactivity but did not enhance reach. This study shows the need to better understand which AMR-specific content factors contribute toward greater traction and to assess audience needs among the wider public to discern how best to harness social media interventions as a tool to improve AMR-related outcomes.

## Introduction

Antimicrobial resistance (AMR) occurs when disease-causing microorganisms (including bacteria, viruses, fungi, and parasites) are no longer affected by antimicrobial agents [1]. In combating the rising threat of AMR, which is expected to result in 8-22 million deaths per year globally by 2050, the World Health Organization (WHO) has emphasized the importance of changing behaviors that contribute to AMR among both the public and health care professionals (HCPs) [2-4].

Global and regional AMR awareness campaigns have been designed to improve public awareness of AMR and inspire related behavioral changes [5]. A common mechanism for delivering such campaigns is through time-specific, coordinated activities that can provide a surge of AMR coverage [6-8]. Such examples include the annual World AMR Awareness Week held in November, and beyond AMR, campaigns such as Mental Health Awareness Week, May Measuring Month (encouraging individuals to measure their blood pressure), and World Cancer Day (advocating for people-centered cancer care) [9-11]. Associated with specific health campaigns, organizations often release content packs or toolkits incorporating key campaign messaging and infographics that can be adopted and shared by individuals and organizations to amplify campaign objectives [12,13].

On the other hand, campaigns such as the United Kingdom Health Security Agency’s “Keep Antibiotics Working” campaign are not designed to be released at a specific time but have nevertheless gathered significant traction and high campaign recognition, including through a social media video with more than 1 million views globally [14]. Other examples of non–time-specific campaigns include the US Food and Drug Administration’s “Little Lungs,” a digital campaign within which a video series delivering antismoking messaging has gathered more than 17 million views on the YouTube platform alone [15].

The past decade has seen great enthusiasm for leveraging the potential of social media as a key vehicle in driving and amplifying health campaigns [12]. Certain HCPs have generated a large network of followers through their active social media presence and have been referred to as “health content creators,” “clinician content creators,” or “health influencers” [16-19]. On the largest video-based social media platform, YouTube, a number of such individuals have gathered more subscribers (individuals who follow their channels and regularly watch their videos) than international and national medical organizations such as the WHO (approximately 874,000 subscribers) and the United States Centers for Disease Control and Prevention (approximately 637,000 subscribers) [20-22]. Increasingly, health organizations have sought to endorse their dedicated networks of health content creators who can serve as credible sources for broadcasting organization-specific health information and tackling misinformation [23]. An example of such a network is the WHO’s network of more than 800 health content creators—WHO Fides [24]—who are mobilized periodically to create content on specific health-related topics and to elevate evidence-based content. Similarly, social media platforms such as TikTok have launched health content creator networks, uniting accredited clinicians to share their insights into trending health topics [25].

Nevertheless, it is unknown how much audience engagement a network of health content creators can generate when they act as messengers for health organizations in broadcasting organization-specific health information. Moreover, it remains uncertain whether delivering the campaign messaging in a time-specific, coordinated manner can generate greater audience traction than the sporadic release of content. Coordinated campaigns have the potential to enhance audience engagement—defined by the number of views, likes, comments, and shares—through repeated exposure, algorithmic amplification, and a sense of social momentum [26,27]. While such a strategy has not previously been explored, it may be a novel avenue for the impactful dissemination of public health information that policymakers and health professionals can use.

In this study, we aimed to answer the question “Can mobilizing an established health content creator network to create social media content on the topic of AMR, released in a coordinated, time-specific manner, have an impact on audience engagement?” Our hypothesis was that a coordinated mobilization of a health content creator network in creating AMR-related social media content can result in greater audience engagement.

## Methods

### Study Design

A longitudinal observational study was conducted to evaluate the impact on audience engagement, when coordinating a health content creator network to disseminate social media videos on the topic of AMR, coinciding with an international event on AMR. We named this coordinated release of social media content “Pulse.”

We first established a health content creator network. A media pack (Multimedia Appendix 1) containing recommended AMR campaign messaging was developed and distributed to the network. Members were informed in advance to prepare AMR-related social media content in accordance with the media pack, to be released on YouTube, the largest video-based social media platform. A specific date was chosen for Pulse to take place, coinciding with a United Nations General Assembly (UNGA) high-level meeting on AMR [28].

The study was reported in accordance with the STROBE (Strengthening the Reporting of Observational Studies in Epidemiology) statement for cohort studies, given the prospective and observational nature of the study [29].

### Study Setting

We conducted the study online on the YouTube (Alphabet Inc) platform, as it is the largest video-based social media platform, and its associated work stream, YouTube Health, has dedicated its mission to delivering high-quality health information online [22]. Registered users of the platform can upload and openly share videos on their YouTube channels. Users can find videos of interest by entering key related terms or phrases into the dedicated YouTube search bar or through the platform’s recommendations. In addition, users can save selected videos into a playlist that can be accessed later. Users can also subscribe to specific channels to receive regular updates. Each video has a unique code (video ID). Each playlist has an ID that is sourced from the web address (also known as URL).

Currently, there are 2 formats of videos on YouTube: traditional long-form videos that individuals browse on a YouTube web page and Shorts, defined as vertical videos that are less than 180 seconds in duration and are presented to users passively and sequentially, from which users can choose either to watch or to swipe away [30].

### Mobilization of a Health Content Creator Network to Release Time-Specific Social Media Video Content on AMR

An international network of health content creators, communications experts, and entertainment professionals was previously created through an in-person launch event on April 29, 2024, and social media platforms such as LinkedIn (Microsoft Corp) and X (X Corp). The network was called CHAIN (Content, Health, and AMR Innovation Network) [31]. The network aims to harness the power and global reach of video-based online content to drive the behavioral changes needed to tackle AMR [31].

A media pack was developed and approved by all researchers over a period of 2 months to coordinate the social media messaging during the Pulse. This media pack was released to CHAIN members immediately following its completion through email 1 month prior to the Pulse date. The media pack incorporated key messages from official sources such as the United Kingdom Health Security Agency and the WHO, explaining what AMR is and how members of the public could change their behaviors related to the development of AMR [32,33].

In addition to key messages, specific messages were created for key niches that the health content creators occupied, including women’s health, children’s health, pharmacists, ear, nose, and throat, dentistry, and medical myths. Creators were given the flexibility in the remaining aspects of video production, such as video format, narration, and imagery, to enable congruency of the content with their existing audience, while maintaining the diversity of content. The media pack is attached in Multimedia Appendix 1.

The Pulse took place on a date that coincided with the UNGA high-level meeting on AMR [28]. Given that this meeting was the principal official, health-focused event during the UNGA high-level meeting week, as well as the second UN high-level meeting on AMR since 2016, we anticipated a heightened global attention toward AMR [34].

### Evaluation of the Impact of the Coordinated Social Media Content Release on Audience Engagement

Six months after the Pulse day, we evaluated the audience engagement (views, likes, and comments) of the content released onto YouTube on the Pulse date using the publicly available engagement data. To discern whether coordinating the social media content had an impact on audience engagement, we undertook comparisons in 2 ways.

First, the engagement analytics of the Pulse videos were compared with those of a similar video that was sporadically released by the same health content creator. A variety of factors can contribute toward the level of engagement of a YouTube video, such as the video content, length of time since video release, thumbnail design and titles, and the channel’s number of subscribers [35]. Choosing a video similar to the Pulse video from the same health content creator allowed for the control of confounding factors, such that a video released in a coordinated manner could be compared against a video released sporadically.

In addition, the engagement of the Pulse videos was compared with the creator’s baseline average video engagement over a 12-month window around the Pulse date. This allowed for direct comparison of the engagement level of the Pulse videos with the average engagement level of the health content creator’s channel, while minimizing time-related confounding factors.

### Data Collection

Following the date of the Pulse, 2 researchers (FC and JC) screened the YouTube channels hosted by members of the CHAIN for all videos released on the date of the Pulse, on the topic of AMR or relating to the appropriate use of antibiotics or other antimicrobial agents. Such videos were saved in a playlist on the YouTube platform. Videos released within 1 day of the Pulse day were also included.

For each Pulse video released by an individual health content creator, a paired video hosted on the same channel was selected by one researcher (FC) and verified by another researcher (KG). When selecting the videos, the following criteria were considered in terms of similarity, ordered by priority: video format (long-form vs Shorts), video topic, recency of release dates, video length, and video style. Given that it was unclear whether the video topic or recency of release can have a greater impact on the level of video engagement, a sensitivity analysis was conducted. An additional video was selected to pair with each Pulse video using slightly different prioritization criteria, in which recency was prioritized over video topic. The paired videos were collated into 2 other YouTube playlists, labeled “paired by topic” and “paired by recency.”

We collected the engagement data of the Pulse videos at the 6-month point from the time of release to allow for the cumulative growth in video engagement to plateau. Therefore, for the paired videos, the researcher collected the engagement analytics at the time point closest to 6 months where possible, although there were variations in the dates of release (eg, some paired videos had been published for 3 years at the time of data collection). The exact dates of data collection for each video are shown in Multimedia Appendix 2.

The engagement analytics were extracted from publicly available YouTube data using YouTube Data application programming interface (API; version 3; Google LLC), which allowed the automated, scaled-up extraction of YouTube data. Specifically, an API key was created on the Google Cloud Platform (Alphabet Inc) and restricted to YouTube Data API [36]. For each playlist (Pulse video, paired by topic and by recency), the specific playlist ID was sourced from the URL of the YouTube playlist. The playlist ID and the API key were then entered into predetermined code using R software (version 4.4.2; R Foundation for Statistical Computing) on the RStudio platform (version 2026.01.0+392; Posit PBC) to extract the data [37,38]. The following data were extracted: YouTube video ID, video title, channel title, video publication date, video duration, video view count, like count, and comment count.

For each health content creator’s videos and engagement data, the channel ID and the date range (a 12-month window around the Pulse date) were entered into the predetermined codes using Python (version 3.12; Python Software Foundation) on VSCode platform (version 1.103; Microsoft Corp) [39,40]. The following data were collected on April 24, 2024: video ID, title, video publication date, views, likes, comments, and video duration.

### Ethical Considerations

Ethics approval for this study was not required as all collected data were publicly available. This was confirmed with the Imperial College London Research Governance and Integrity Team in accordance with local policies [41]. All data have been anonymized and deidentified.

Informed consent was not obtained because the study analyzed publicly accessible online content. In accordance with relevant ethical guidance for internet-based research, the study involved minimal risk and did not include any attempt to identify or contact individuals whose content was analyzed. Participant compensation was not applicable.

### Statistical Analysis

Summary statistics were calculated for the Pulse videos, including engagement data and video duration. Engagement outcomes (views, likes, and comments) were analyzed using a staged analytical approach. First, unadjusted comparisons were conducted between Pulse videos and noncoordinated videos paired by topic from the same health content creator. To assess for normality, differences in engagement were first computed within each pair, and the difference scores were assessed using the Shapiro-Wilk test [42]. For metrics for which the assumption of normality was met (*P*>.05), a paired 2-tailed *t* test was performed [43]. For metrics that violated the normality assumption (*P*≤.05), the Wilcoxon signed-rank test was used as a nonparametric alternative [44]. Results were presented as the Hodges-Lehmann median differences with 95% CIs [45]. Second, to account for residual confounding, adjusted analyses were conducted using negative binomial regression [46].

Engagement data were modeled as count outcomes, with Pulse status specified as the primary exposure. Models were adjusted a priori for video-level characteristics influencing engagement, including video format (Shorts vs long-form), video duration, and time since publication at the point of data extraction (expressed in months). To account for correlation of outcomes within creators, robust SEs clustered at the creator level were used. Results from regression analyses were presented as incidence rate ratios (IRRs) with 95% CIs. Statistical significance was set at *α*=.05.

To evaluate how the Pulse videos compared with each creator’s average performance, we calculated the median and IQR of engagement metrics (views, likes, and comments) for each creator’s noncoordinated videos published within this 12-month interval. To assess normality, differences were calculated for each creator’s Pulse video and median engagement of the baseline videos and were assessed using the Shapiro-Wilk test [42]. Given the anticipated highly skewed distribution of engagement metrics and the paired within-creator design, differences in views, likes, and comments between the Pulse videos and the median engagement values of the same creator were analyzed using the Wilcoxon signed-rank tests. In addition, the Pulse videos were compared with the mean performance of videos of the same format (long-form or Shorts) within the 12-month window around the coordinated release date, using the same method described. The Hodges-Lehmann median differences and 95% CIs were reported. Statistical significance was set at *α*=.05.

Data collection and analysis were conducted in R software on the RStudio platform [37,38]. The following packages were used: *httr*, *httr2*, *jsonlite*, *here*, *dplyr*, *vosonSML*, *readxl*, *ggplot2*, *effsize*, *tidyverse*, *reshape2*, *MASS*, *sandwich*, *lmtest*, and *broom* [38,40,47-59].

## Results

### Overview

Around the Pulse date on September 26, 2024, the health content creator network released 18 videos from 14 YouTube channels hosted by members of the network. Six months after the Pulse, the audience engagement data for all 18 coordinated videos (V1-V18) were successfully collected, with no missing data. Overall, the coordinated videos gathered a total of 6153 views, 262 likes, and 76 comments. The campaign media pack and the complete video engagement datasets are available in MultimediaAppendices 1  2, respectively.

### Evaluating the Impact of a Coordinated Release on Video Engagement

#### Comparison of the Pulse Videos With Paired Videos

The chosen paired videos prioritizing topic choice (P1-P18) and recency (R1-R18) are shown in Multimedia Appendix 2. All engagement analytics were successfully collected with no missing data. The summary characteristics of the 3 video groups (Pulse, paired by topic, and paired by recency) are shown in Table 1 and Table S1 in Multimedia Appendix 3. Assessment of normality using the Shapiro-Wilk normality test is shown in Table S2 in Multimedia Appendix 3.

**Table 1.** Summary characteristics of the “Pulse” videos and the paired videos by the same health content creators^a^.

Engagement metrics	Pulse videos, median (IQR)	Paired by topic, median (IQR)	Hodges-Lehmann estimator, median difference (95% CI)	*P* value
View count	123 (39-580)	72 (36-367)	58.5 (–31.5 to 237.0)	.15
Like count	10 (4-31)	7 (2-12)	8.0 (0.5-19.0)	.04
Comment count	2 (1-5)	1 (0-2)	2.0 (1.0-7.0)	.04
Duration (s)	95 (60-247)	134 (59-238)	—^b^	—
Time since publication (mo)	6 (6-6)	6.5 (6-15)	—	—

a The paired videos were chosen by prioritizing video topics. Paired differences in engagement (views, likes, and comments) were assessed using the Wilcoxon signed-rank test.

b Not applicable.

The differences in engagement between the video pairs were assessed using the Wilcoxon signed-rank test. When comparing the Pulse videos (V1-V18) with the videos paired by topic (P1-P18), no significant difference was found between the view counts (IRR 58.5, 95% CI –31.5 to 237.0; *P*=.15). However, the Pulse videos appeared to have significantly more like counts (IRR 8.0, 95% CI 0.5-19.0; *P*=.04) and comment counts (IRR 2.0, 95% CI 1.0-7.0; *P*=.04). Sensitivity analysis pairing videos by recency showed concordant findings (Table S1 in Multimedia Appendix 3).

After adjustment for confounding (Table 2), Pulse videos were not associated with higher view counts or like counts but were associated with significantly higher comment counts (IRR 2.99, 95% CI 1.02-8.52; *P*=.03). In sensitivity analyses pairing videos by recency rather than topic (Table S3 in Multimedia Appendix 3), effect estimates were directionally consistent, although associations were attenuated and no longer statistically significant.

**Table 2.** Adjusted association between “Pulse” videos and audience engagement^a^.

Association	Views		Likes		Comments	
	IRR^b^ (95% CI)	*P* value	IRR (95% CI)	*P* value	IRR (95% CI)	*P* value
Pulse (vs noncoordinated)	0.98 (0.44-2.13)	.95	1.10 (0.48-2.41)	.81	2.99 (1.02-8.52)	.03
Shorts (vs long-form content)	7.79 (2.98-20.49)	<.001	4.81 (1.97-11.93)	<.001	1.42 (0.45-4.53)	.51
Video duration (per 60 s)	1.06 (0.92-1.25)	.35	1.01 (0.88-1.20)	.82	1.00 (0.81-1.25)	.99
Time since publication (per mo)	1.03 (0.97-1.19)	.40	1.00 (0.93-1.07)	.93	1.00 (0.91-1.11)	.97

a Negative binomial regression models were used. Models were adjusted for video format, video duration, and time since publication. Robust SEs clustered at the creator level were used to account for within-creator correlation. Videos were paired based on topics.

b IRR: incidence rate ratio.

#### Comparison of the Pulse Video With the Creator’s Average Level of Engagement

The summary characteristics of all creators’ baseline videos are shown in Multimedia Appendix 2. When comparing the Pulse videos with each creator’s baseline performance, there was no significant difference in the number of views (IRR −82.0, 95% CI −190.3 to 58.5; *P*=.26) or like counts (IRR −0.50, 95% CI −6.3 to 10.5; *P*=.93). However, the Pulse videos had a significantly greater number of comments (IRR 15.5, 95% CI 5.5-24.0; *P*<.001; Table 3). When comparing the Pulse videos against creators’ baseline videos of the same format, no significant differences were observed (Table S4 and S5 in Multimedia Appendix 3).

**Table 3.** “Pulse” videos compared with creator’s baseline videos released over a 12-month window around the Pulse date^a^.

Engagement metrics	Hodges-Lehmann estimator, median difference (95% CI)	*P* value
View count	−82.0 (−190.3 to 58.5)	.26
Like count	0.50 (−6.3 to 10.5)	.93
Comment count	15.5 (5.5 to 24.0)	<.001

a Values are shown as Hodges-Lehmann median difference (95% CI). Paired differences in engagement (views, likes, and comments) were assessed using the Wilcoxon signed-rank test.

## Discussion

### Principal Findings

This study examined whether coordinating health content creators to release AMR-related videos simultaneously on YouTube, timed with an AMR-centered international event, could enhance audience engagement compared to sporadic, uncoordinated releases. The principal findings were that coordinating health content creators to release AMR-related videos on YouTube coinciding with an international event on AMR, defined as a Pulse, increased audience interactivity in terms of the number of comments but did not enhance overall reach compared with sporadic releases.

When comparing the Pulse videos with similar videos and baseline videos from the same health content creator, coordinated content gathered significantly greater comment counts. This suggests that coordinated campaigns may enhance interactive engagement among audiences, potentially through the perception of collective momentum or amplified algorithmic exposure. However, the lack of a significant difference in view counts and like counts after adjustment between coordinated and sporadic videos suggests that coordination alone may not be sufficient to drive greater overall reach.

It is noteworthy that Shorts, compared with long-form videos, have been found to be significantly associated with greater engagement in terms of views and likes (*P*<.001). While YouTube has traditionally been the main hub for long-form video content, the introduction of YouTube Shorts has transformed how individuals consume social media content. The audience tends to spend more time watching shorter videos, which provides them with bite-sized content, while long-form videos have seen a significant decrease in engagement [60].

Several possible explanations exist for the principal findings. First, although coordination can enhance visibility within a narrow window, it does not necessarily equate to broader audience interest or long-term viewership. Second, the relatively small size of the coordinated cohort (18 videos across 14 channels) may not have generated the critical mass required to trigger meaningful algorithmic amplification. Third, the media pack was developed based on accuracy of messaging rather than the enhancement of audience engagement. Currently, there remains a lack of research on how to design video content, particularly AMR-specific content, to improve audience engagement.

From a campaign design perspective, these findings suggest that while coordination can enhance engagement quality and audience interactivity (as seen in likes and comments), it may not inherently improve reach. Therefore, coordinated campaigns may be best seen as complementary tools within a broader health communication strategy rather than as standalone solutions.

Previous HCP-led social media campaigns have mostly aimed at HCPs, focusing on advocacy for particular specialties such as surgery, radiology, and nursing [61-64]. The remaining HCP-led social media campaigns took place during the COVID-19 pandemic, relating to vaccination and travel restrictions [64-66]. No studies have evaluated the impact of collaborating with HCPs in delivering time-coordinated social media campaigns relating to other global health challenges outside the COVID-19 pandemic period. To our knowledge, this study is the first evaluation of the impact of collaboration with health content creators during an international public health campaign. Our findings provide important insights into the dynamics of health communication on social media platforms and the potential role of health content creator networks in supporting public health campaigns [67,68].

### Limitations

Several limitations should be acknowledged. The sample size of creators and videos was relatively small, limiting statistical power and generalizability. The observational design precludes causal inference. While we matched video pairs based on format, topic, and recency, not all engagement data were collected at the 6-month point from their time of release. Some creators posted relatively infrequently, such that their most recent video was posted 24 months ago. Although we welcomed creators internationally, most participating health content creators were based in the United Kingdom, meaning that the health challenge of AMR was competing with other country-specific challenges. Moreover, engagement metrics such as views, likes, and comments do not necessarily reflect a deeper impact on audience such as changes in knowledge, changes in attitudes, or AMR-related behavioral changes, which were not explored in this study.

### Opportunities for Future Work

Future work should explore how to optimize coordinated health campaigns on social media platforms to support health policymakers in designing more effective public health strategies. This may include testing different messaging styles, content formats, and durations of such coordinated activity. Second, it will be valuable to assess the impact of such campaigns on AMR-related outcomes such as individual knowledge, attitudes, and behavioral intentions, which can be collected from YouTube through qualitative analysis of the audience comments, as well as through embedded survey methodologies as part of the campaign content. Finally, the study calls for the need to better understand which AMR-specific content factors contribute toward greater audience traction and for assessment of audience needs among the wider public, to discern how best to harness social media interventions as a tool to improve AMR-related outcomes, including inspiring behavioral changes.

### Conclusions

This study highlights both the potential and the limitations of using social media to engage the public on AMR. While campaigns led by health content creators increased audience interactivity, they did not substantially improve overall reach, reflecting the broader challenge of promoting AMR-related content in crowded social media environments. These findings highlight the need to understand the nuances behind audience behavior, preferences, and engagement dynamics within social media platforms, such that the content can objectively influence knowledge, attitudes, and behaviors. Importantly, our results underscore the need for innovative evaluation methods that capture the real-world impact of social media campaigns on AMR-related outcomes. By integrating content strategy with behavioral insights, future campaigns could more effectively leverage digital platforms to support antimicrobial stewardship and inform policy on health communication in the social media era.

## Supplementary material

10.2196/86587 Multimedia Appendix 1 Antimicrobial resistance campaign media pack.

10.2196/86587 Multimedia Appendix 2 Pulse videos and paired videos selected by topic and by recency.

10.2196/86587 Multimedia Appendix 3 Supplementary tables.

## Abbreviations

AMR: antimicrobial resistance
API: application programming interface
CHAIN: Content, Health, and Antimicrobial Resistance Innovation Network
HCP: health care professional
IRR: incidence rate ratio
STROBE: Strengthening the Reporting of Observational Studies in Epidemiology
UNGA: United Nations General Assembly
WHO: World Health Organization
