# Supplementary material for: The Impact on Audience Engagement of Coordinating a Public Health Campaign on Antimicrobial Resistance Through a Network of Health Content Creators: Longitudinal Observational Study
Source: JMIR Public Health Surveill. 2026 Mar 27;12:e86587. doi: 10.2196/86587 (PMC13027679; doi:10.2196/86587)
Supplement: Multimedia Appendix 1 [file publichealth-v12-e86587-s001.pdf]

FLEMING INITIATIVE

SEP 26 2024

15:00 BST

10:00 EST

CHAIN PULSE TOOLKIT

COORDINATING VIDEO CONTENT FOR THE UN GENERAL ASSEMBLY HIGH-LEVEL MEETING ON ANTIMICROBIAL RESISTANCE

FLEMING INITIATIVE

IMPERIAL

NHS
Imperial College Healthcare
NHS Trust

CONTENTS

Welcome from the Fleming Initiative....... 4

What is antimicrobial resistance?...........6

CHAIN Pulse: What, where, when, why...7

CHAIN Pulse: Our asks..........................8

Why take part?....................................12

Core messages....................................13

Message for women's health................16

Messages for children's health.............18

Message for pharmacists......................20

Message for ENT..................................20

Messages for dentistry..........................21

Messages for medical myths................22

Brand guidelines..................................23

References..25

Welcome from the Fleming Initiative

Dear CHAIN members,

Thank you for your engagement with our network since its launch at the House of Lords. We are lucky to be working with such multi-faceted creators, many of you producing trustworthy health-focused video content in addition to your usual clinical work.

The World Health Organization has declared antimicrobial resistance (AMR) as one of the top 10 global threats to public health [1]. If we don't tackle this problem, drug-resistant infections have the potential to become a global humanitarian crisis, responsible for at least 10 million lives lost yearly by 2050: more than cancer [2].

At the Fleming Initiative, we would like to recognise your skill and influence by bringing you into the very centre of our efforts against antimicrobial resistance.

We must raise awareness of antimicrobial resistance – its causes and solutions – to drive positive global change.

And we must change how people – the public and health professionals – understand and use antimicrobials, using behavioural science-informed interventions.

We are asking you to take part in the **CHAIN Pulse: a coordinated release of AMR video content** set to coincide with the United Nations General Assembly High-Level Meeting on AMR on September 26th.

The High-Level Meeting on AMR will be convened in New York City and the Fleming Initiative will be there on the ground raising awareness amongst the public, policymakers, and media.

This is a rare opportunity where the world's eyes will be on the issue of AMR and the Fleming Initiative, and a great opportunity for you to leverage your skills, knowledge, and influence to raise awareness of AMR and promote your platform to new audiences.

You can reach people all around the world with trustworthy, authoritative health information.

You will to able to reach people the Fleming Initiative never could, and connect them to key messages around antimicrobial resistance in ways we can't.

We hope you will help make the CHAIN Pulse a success. As ever, don't hesitate to reach out to info@fleminginitiative.org with any questions or suggestions.

Reference 1: WHO, Ten threats to global health in 2019

Reference 2: WHO, No Time to Wait: Securing the Future from Drug-Resistant Infections

What is antimicrobial resistance?

Antimicrobials are medicines used to prevent and treat infections in humans, animals, and plants. The microbes that cause infection can develop resistance to these medicines.

Widespread misuse and overuse of antibiotics and other antimicrobials has led to the global spread of drug-resistant microbes known as antimicrobial resistance (AMR).

AMR is one of the top global public health threats. It is estimated that bacterial AMR was directly responsible for 1.27 million global deaths in 2019 and contributed to 4.95 million deaths [2].

If we don't tackle this problem, drug-resistant infections have the potential to become a global humanitarian crisis, responsible for at least 10 million lives lost yearly by 2050: more than cancer [2].

Without action, we are heading for a post-antibiotic era, where a common infection or routine surgical procedure could become life threatening.

It is possible to control the spread of drug-resistance, but medical breakthroughs alone will not be the solution to this invisible health crisis.

Science must work hand-in-hand with policy and people to deliver meaningful change.

CHAIN Pulse: What, where, when, why

WHAT

The Fleming Initiative will mobilise CHAIN members to deliver a powerful, coordinated video message that will put the spotlight on fighting antimicrobial resistance through behavioural change, coinciding with the UN General Assembly (UNGA) High-Level Meeting on Antimicrobial Resistance in New York City.

WHERE

YouTube is our priority platform, but we encourage video content to be posted and promoted on other platforms, including Instagram, TikTok, X/Twitter, and LinkedIn.

WHEN

Thursday September 26th, 15:00 UK time (10:00 EST)

WHY

The world's attention will be on UNGA and the problem of antimicrobial resistance. The CHAIN Pulse will take advantage of the global attention that the Fleming Initiative will receive as they participate in the UNGA: this is a rare opportunity to leverage your knowledge, skills, and influence in the fight against antimicrobial resistance.

CHAIN Pulse: Our asks

FORMAT

We welcome both YouTube Shorts and regular YouTube content equally.

Any video length is welcomed.

Please post CHAIN Pulse content on your own channels.

CONTENT

We ask that you incorporate the messages laid out in this toolkit's 'Core messages for the CHAIN Pulse' section, which were selected to raise awareness of what AMR is and what your audiences can do to fight its spread.

You may wish to expand on these by incorporating further messages detailed in later sections of this toolkit.

KEY LANGUAGE AND VIDEO TITLE

Please prioritise the term 'antimicrobial resistance'. You may also wish to use the terms 'drug resistant infections' and 'antibiotic resistance'.

We would encourage the inclusion of 'AMR' or 'antimicrobial resistance' in the video title.

"What is AMR" and "How does AMR occur" are the 2 most commonly searched queries on YouTube related to AMR over the past several years.

REQUIRED TEXT FOR VIDEO DESCRIPTIONS

Please include a link to our website (www.fleminginitiative.org) and the hashtag #fleminginitiative

REQUIRED HASHTAG FOR SOCIAL MEDIA POSTS

#fleminginitiative

OPTIONAL HASHTAGS

#UNGA #KeepAntibioticsWorking
#AMR #CHAIN #antimicrobialresistance
#antibioticresistance

OPTIONAL BRANDING

If possible, we would appreciate if you could please:

1) use the Fleming Initiative logo at an appropriate point in the video content

2) mention the Fleming Initiative by name

3) draw from the specific language detailed in the Branding section of this toolkit, if you do want to talk about the Fleming Initiative's mission.

ACCESS TO ANALYTICS

We would like to evaluate the impact of the CHAIN Pulse over time.

This analysis will form part of a body of research being conducted at the Fleming Initiative to explore the real world impact of online health content.

In order to do so, we would kindly request that you provide us with the performance analytics of your CHAIN Pulse content, 1 week, 1 month, and 3 months after the Pulse.

We will assess the effectiveness of a coordinated video message strategy for raising awareness of antimicrobial resistance, and feedback the results of our analysis to you, with individual and CHAIN-wide insights.

This would inform future campaigns by the Fleming Initiative and we hope that through our co-location with policy makers and international partners, its reach will be felt further afield.

TAG AND INFORM US

Let us know if you plan on taking part!

Please consider letting us know via email (info@fleminginitiative.org) so that we can plan to share your content from Fleming Initiative, Imperial College London, and Imperial College Healthcare NHS Trust social media accounts and with internal networks.

You may tag Imperial College London and Imperial College Healthcare NHS Trust accounts, but please prioritise the Fleming Initiative. If you do tag these organisations, both should be tagged.

All organisations will promote and engage with CHAIN Pulse content.

TAG US ON X/TWITTER

@FlemingCentre
@imperialcollege
@imperialNHS

TAG US ON LINKEDIN

Fleming Initiative
Imperial College London
Imperial College Healthcare NHS Trust

TAG US ON INSTAGRAM

@fleminginitiative
@imperialcollege
@imperialNHS

TAG US ON YOUTUBE

@FlemingInitiative
@imperialcollegevideo
@imperialcollegehealthcaren1020

Why take part?

MAKE A DIFFERENCE

The UK government's call for evidence on AMR found that there is insufficient public awareness of AMR (95% of respondents).

We must raise awareness of antimicrobial resistance – its causes and solutions – to drive positive global change. And we must change how people – the public and health professionals – understand and use antimicrobials, using behavioural science-informed interventions.

Video content and clinician creators such as yourselves are perfectly placed to further these goals. You can reach people all around the world with trustworthy, authoritative health information – you can reach people The Fleming Initiative never could, and connect them to key messages around antimicrobial resistance in ways we can't.

GLOBAL ATTENTION

The CHAIN Pulse will take advantage of the attention that the issue of AMR and the Fleming Initiative itself will receive from media and members of the public around the world, as the UN General Assembly High-Level Meeting on AMR happens.

This is a rare opportunity where the world's eyes will be on the issue of AMR, and a great opportunity for you to leverage your skills, knowledge, and influence to raise awareness of AMR and promote your platform to new audiences

INCENTIVES

The Fleming Initiative will gift £50 Amazon vouchers to the creators who produced the a) best performing video in terms of viewer numbers, b) the most creative video, and c) the video with most engagement.

We will also offer an exclusive dinner in central London in early 2025 to creators who take part in the CHAIN Pulse, during which you will receive early access to campaign insights.

Core messages for the CHAIN Pulse

MESSAGE ONE

We can control the spread of antimicrobial resistance and keep antibiotics working, but everyone needs to make behavioural changes to combat its causes – to ensure antimicrobials are used appropriately.

Please find examples below of behavioural changes that you can suggest to your audiences, to help them fight the spread of antimicrobial resistance when taking antimicrobials.

- If you or anyone in your family are prescribed antibiotics, take them exactly as prescribed and never save them for later or share them with others. As part of this, ensure you finish the prescribed course of antibiotics. Stopping your prescribed course early could allow some of the bacteria to remain in the body, reproduce, and emerge as a resistant strain.

- For infections like coughs, colds, sore throats and flu, which our bodies are good at fighting off on their own, you should talk to a pharmacist about how to treat the symptoms first rather than going to the GP. We want to ensure antibiotics are used at the right time, for the right indications.

- Don't self-medicate with leftover antibiotics or those obtained without a prescription.

We are **not** saying that we should all stop using antimicrobials – they are an essential component of our healthcare – we are saying that they should only be used **when** appropriate, and that they should **be used** appropriately.

MESSAGE TWO

We depend on antimicrobials for so much more than we realise. Making behavioural changes to prevent the spread of antimicrobial resistance doesn't only protect you, it means you are helping to protect the most vulnerable members of society.

Antimicrobials are at the heart of how we treat infectious diseases. They make childbirth safer, allow safe surgery, protect the immunosuppressed (such as cancer patients), and support the health and welfare of our pets and livestock.

As drug resistance spreads, common infections and injuries that were once easily treatable become harder, and in some cases impossible, to treat.

Please find examples of behavioural changes you can suggest to your audiences to help them fight the spread of antimicrobial resistance every day, even when they're not unwell.

- By taking small steps everyday to maintain good personal hygiene, you are helping to prevent the spread of infection and protecting yourself and those around you from contracting a drug resistant infection, or an infection that might later develop drug resistance.
- Cover your coughs and sneezes with a tissue or your sleeve, clean frequently touched surfaces, cover all cuts and abrasions with a waterproof dressing, and wash or sanitise your hands often (always after going to the toilet, before eating or preparing food, and after touching animals) [3].
- The World Health Organization has identified safe drinking water, sanitation, and hygiene (WASH) as being key to preventing infections and reducing the spread of AMR [4].

Reference 3: UK Health Security Agency Guidance, Preventing and Controlling Infections

Reference 4: WHO, WASH and antibiotic resistance

OPTIONAL MESSAGE THREE

The Fleming Initiative recognises that it is possible to control the spread of antimicrobial resistance, but medical breakthroughs alone will not be the solution to this invisible health crisis.

- The Fleming Initiative offers an innovative approach to the challenge of AMR – co-locating research, behaviour change, public engagement, and policy to provide real-world solutions that work to protect the health of local populations around the world.
- Prevention is better than cure – we need to develop sustainable solutions before mortality soars. The time to act is now.

WHAT DOES THE PUBLIC WANT TO SEE?

Our research analysing the current YouTube content on AMR found that the most popular videos on AMR described to the public: 1) what AMR is, 2) how antibiotics work, 3) and how antimicrobial resistance can stop them working.

See the following sections for additional messages on clinical subjects and specialisms that are well represented amongst our CHAIN members. You may wish to supplement our core messages with these additional messages.

Message for women's health

Urinary tract infections (UTIs) disproportionately affect women at all stages of life, and are one of the leading reasons for antibiotic prescriptions worldwide.

Recurrent UTIs are common. If you experience recurrent UTIs, do not take leftover tablets from previous antibiotic prescriptions, consult with your pharmacy, GP, etc.

You may have a drug resistant infection that requires treatment with a different antimicrobial, your previous prescription may be out-of-date and no longer effective, and you will likely not have enough pills from your previous prescription to complete a full course of treatment.

Messages for children's health

Infants are more susceptible to drug resistant infections than adults because a) their immune systems are not fully developed [5] and b) they interact with their environment differently to adults (exposure to microbes via crawling on the ground, placing objects in their mouths etc).

Children living in poverty are even more susceptible to drug resistant infections, for reasons such as lack of access to safe drinking water, sanitation, and hygiene. Among children under five who die from AMR, 99.65 percent are in low- or middle-income countries [6].

254,000 children under five die from AMR each year, meaning a child dies of AMR every two minutes [6].

MESSAGE ONE

Parents, caregivers, educators, and community leaders can promote positive behaviour change amongst children and young people by using the evidence-based, Association for Science Education accredited, and UKHSA-operated online e-Bug resources.

There are resources and lesson plans to teach about hygiene, microbes, vaccinations, and antimicrobial resistance from ages 3-16 at www.e-bug.eu/teachers

Reference 5: Proc Biol Sci, Evolution of the immune system in humans from infancy to old age

Reference 6: The Lancet, Global burden of bacterial antimicrobial resistance in 2019: a systematic analysis

MESSAGE TWO

In addition to the actions highlighted in our core messages, vaccinations are important in safeguarding children against drug-resistant infections.

Beyond protecting an individual child from infection, vaccines can reduce antibiotic misuse, therefore reducing the emergence and transmission of drug resistant infections.

The 6-in-1 and pneumococcal vaccines are important in preventing infections by Haemophilus influenzae type b (Hib) and bacterial infections that cause pneumonia, and have a great track record for preventing the emergence and transmission of drug resistant infections [7, 8].

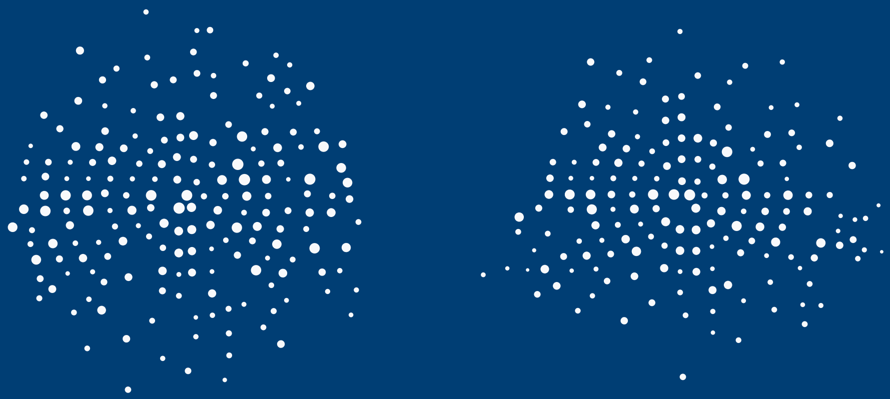

Reference 7: NHS Vaccinations and when to have them

Reference 8: Hum Vaccin Immunother, The role of vaccines in fighting antimicrobial resistance (AMR)

Message for pharmacists

The 2015 WHO Multi-Country Public Awareness Survey on Antimicrobial Resistance found that nearly a third of people (32%) believe they should stop taking antibiotics when they feel better, rather than completing the prescribed course of treatment [9].

We believe the public could benefit from increased messaging around completing the prescribed course of treatment, with pharmacists being a trustworthy and impactful group to communicate this messaging.

Reference 9: WHO, Antibiotic resistance: multi-country public awareness survey

Message for ENT

In England and globally, respiratory tract infections are the leading reason for prescribing antibiotics [10, 11]. In England, antibiotic treatments for upper respiratory tract indications account for more than two thirds of total prescriptions [11]. Most ENT infections, like the common cold and viral sinusitis, do not require antibiotics.

Primary care professionals working in ENT are therefore very well placed to have an impact on preventing the spread of antimicrobial resistance by raising awareness of AMR and appropriate behavioural changes to prevent its spread amongst patients and the public.

Appropriate behavioural changes include a) those discussed in 'Core messaging for the CHAIN Pulse' and b) recommend alternative treatments for symptom relief, such as analgesics, decongestants, saline nasal sprays, and steam inhalation for viral infections.

Reference 10: Ther Adv Drug Saf, Antimicrobial resistance: risk associated with antibiotic overuse and initiatives to reduce the problem

Reference 11: BMJ, Duration of antibiotic treatment for common infections in English primary care: cross sectional analysis and comparison with guidelines

Messages for dentistry

MESSAGE ONE

Dental professionals are responsible for ~ 10% of antibiotics prescriptions in the UK and globally [12, 13]. Given this, messaging from dental professionals to patients around the appropriate use of antibiotics is particularly impactful.

Reference 12: [FDI World Dental Federation, Antibiotic Stewardship in Dentistry]

Reference 13: [Int Dent J, Tackling Antibiotic Resistance: Why Dentistry Matters]

MESSAGE TWO

Preventing dental infections, such as through oral hygiene and dietary advice, is important in preventing the spread of drug resistant infections. Dental professionals are best placed to raise awareness of best dental prevention practices.

Messages for medical myths

MYTH ONE

In antimicrobial resistance, it is your body that becomes resistant to antimicrobials.

- No – in antimicrobial resistance, it is the microbes that have developed resistance to antimicrobials.
- This is important in understanding the reasoning behind behavioural changes that can help us fight the spread of antimicrobial resistance, such as completing your prescribed course of antibiotics.
- The 2015 WHO Multi-Country Public Awareness Survey on Antimicrobial Resistance found that 76% of respondents believed this myth [9].

MYTH TWO

You can only contract a drug resistant infection if you take antimicrobials.

- No – It is possible for someone to contract a drug resistant infection even if they've never taken antibiotics. A drug resistant infection can be caught in many ways, including transmission from other people or contaminated surfaces.
- This is important in understanding the reasoning behind behavioural changes that can help us fight the spread of antimicrobial resistance by preventing infection, such as vaccination and good hygiene practices.
- The 2015 WHO Multi-Country Public Awareness Survey on Antimicrobial Resistance found that 44% of respondents believed this myth [9].

Brand guidelines

LANGUAGE

When named, Imperial College London and Imperial College Healthcare NHS Trust must be named in full. For instance, do not say 'Imperial' by itself.

Suggested language regarding the Fleming Initiative:
"The Fleming Initiative is a partnership between Imperial College London and Imperial College Healthcare NHS Trust"

If mentioned, CHAIN may be referred to as 'CHAIN', 'the CHAIN network', or 'the Content, Health, and AMR Innovation Network'.

LOGO

If the Fleming Initiative logo is featured at a relevant point in your video content, a minimum exclusion space around the logo equivalent to half the logo's height is required.

You can download high-resolution logo files here with this exclusion space applied.

OPTIONAL COPY

Please consider including the following text in your video description.

If further context on the Fleming Initiative is provided within the video itself, the following text may be used as presented or paraphrased:

Founded on a partnership between Imperial College London and Imperial College Healthcare NHS Trust, the Fleming Initiative is bringing research, behaviour change, public engagement, and

policy together to provide real-world solutions to a global problem.

It is possible to control the spread of drug-resistance, but medical breakthroughs alone will not be the solution to this invisible health crisis.

Science must work hand-in-hand with policy and people to deliver meaningful change.

The Fleming Initiative will break down barriers between scientists, policymakers, and the public through the founding of The Fleming Centre, a co-location space at St Mary's Hospital, where Sir Alexander Fleming discovered penicillin in 1928.

In this innovative new ecosystem – where clinicians work side by side with microbiologists, AI experts, behavioural scientists and policymakers, and all connect with the public – powerful new ideas will emerge and thrive.

Learn more at www.fleminginitiative.org

If further context on CHAIN is provided, the following text may be used as presented or paraphrased.

The Content, Health and AMR Innovation Network (CHAIN) is a new media network led by The Fleming Initiative, with a membership of clinician creators, communications experts, and entertainment professionals.

CHAIN aims to harness the power and global reach of video-based online content to drive the behaviour changes needed to tackle the spread of drug-resistant microbes known as antimicrobial resistance.

References

Reference 1: WHO, Ten threats to global health in 2019

Reference 2: WHO, No Time to Wait: Securing the Future from Drug-Resistant Infections

Reference 3: UK Health Security Agency Guidance, Preventing and Controlling Infections

Reference 4: WHO, WASH and antibiotic resistance

Reference 5: Proc Biol Sci, Evolution of the immune system in humans from infancy to old age

Reference 6: The Lancet, Global burden of bacterial antimicrobial resistance in 2019: a systematic analysis

Reference 7: NHS Vaccinations and when to have them

Reference 8: Hum Vaccin Immunother, The role of vaccines in fighting antimicrobial resistance (AMR)

Reference 9: WHO, Antibiotic resistance: multi-country public awareness survey

Reference 10: Ther Adv Drug Saf, Antimicrobial resistance: risk associated with antibiotic overuse and initiatives to reduce the problem

Reference 11: BMJ, Duration of antibiotic treatment for common infections in English primary care: cross sectional analysis and comparison with guidelines

Reference 13: FDI World Dental Federation, Antibiotic Stewardship in Dentistry

Reference 13: Int Dent J, Tackling Antibiotic Resistance: Why Dentistry Matters

FLEMING INITIATIVE

SEP 26 2024

15:00 BST

10:00 EST

CHAIN PULSE TOOLKIT

WRITTEN AND DESIGNED BY JACK COOPER

IMPERIAL

NHS Imperial College Healthcare NHS Trust
