# Supplementary material for: The Impact on Audience Engagement of Coordinating a Public Health Campaign on Antimicrobial Resistance Through a Network of Health Content Creators: Longitudinal Observational Study
Source: JMIR Public Health Surveill. 2026 Mar 27;12:e86587. doi: 10.2196/86587 (PMC13027679; doi:10.2196/86587)
Supplement: Multimedia Appendix 3 [file publichealth-v12-e86587-s003.docx]

**Multimedia Appendix 3**

|  | Pulse videos | Videos Paired by recency | Hodges-Lehmann Median difference (95% CI) | P-value |
| --- | --- | --- | --- | --- |
| View count | 123 (39; 580) | 126 (41; 415) | 60 (-9; 193) | 0.09 |
| Like count | 10 (4; 31) | 6 (1; 12) | 10 (0.5; 21) | 0.04 |
| Comment count | 2 (1; 5) | 1 (0; 2) | 2 (0.500; 7) | 0.04 |
| Duration in seconds | 95 (60; 247) | 118 (59; 397) |  |  |
| Time since publication (months) | 6 (6; 6) | 6 (6; 8) |  |  |

**Supplementary Table 1.** Summary characteristics of the “Pulse” videos and the paired videos by the same health content creators. The paired videos were chosen prioritizing video recency. Values are shown as median (Interquartile range) or Hodges-Lehmann Median difference (95% Confidence Interval). Paired differences in engagement (views, likes and comments) were assessed using Wilcoxon Signed rank test. CI: confidence interval.

| **Parameters** | **W statistic** | **P-value** |
| --- | --- | --- |
| Difference in view count | 0.65 | <0.001 |
| Difference in Like count | 0.77 | <0.001 |
| Difference in comment count | 0.77 | <0.001 |

**Supplementary Table 2.** Assessment of normality using Shapiro-Wilk normality test of difference in engagement counts between the “Pulse” video and selected paired videos.

|  | Views | | Likes | | Comments | |
| --- | --- | --- | --- | --- | --- | --- |
|  | IRR (95% CI) | *P* value | IRR (95% CI) | *P* value | IRR (95% CI) | *P* value |
| Pulse (vs non-coordinated) | 0.78 (0.39; 1.52) | 0.44 | 1.06 (0.48; 2.28) | 0.87 | 2.07 (0.69; 5.90) | 0.14 |
| Shorts (vs long-form) | 4.90 (2.48; 9.82) | <0.001 | 4.26 (1.98; 9.27) | <0.001 | 1.24 (0.47; 3.36) | 0.66 |
| Video duration (per 60 seconds) | 1.00 (0.98; 1.04) | 0.97 | 1.01 (0.98; 1.05) | 0.55 | 1.02 (0.98; 1.09) | 0.27 |
| Time since publication (per month) | 0.90 (0.83; 0.99) | 0.013 | 0.94 (0.84; 1.05) | 0.24 | 0.79 (0.48; 1.02) | 0.14 |

**Supplementary Table 3.** Adjusted association between “Pulse” videos and audience engagement. Negative binomial regression models were used. Models were adjusted for video format, video duration, and time since publication. Robust standard errors clustered at the creator level were used to account for within-creator correlation. Videos were paired based on recency. IRR: Incidence Rate Ratios; CI: Confidence Intervals.

|  | Hodges-Lehmann Median difference (95% CI) | *P*-value |
| --- | --- | --- |
| View count | -6.22 (-116; 137) | 0.93 |
| Like count | 2.50 (-7; 14) | 0.44 |
| Comment count | 1.5 (-0.25; 7.25) | 0.09 |

**Supplementary Table 4.** “Pulse” videos compared with creator’s baseline videos matched by the same format. Values are shown as Hodges-Lehmann Median difference (95% Confidence Interval). Paired differences in engagement (views, likes and comments) were assessed using Wilcoxon Signed rank test. CI: confidence interval.

| **Parameters** | **W statistic** | **P-value** |
| --- | --- | --- |
| Difference in view count | 0.92 | 0.11 |
| Difference in Like count | 0.90 | 0.047 |
| Difference in comment count | 0.83 | 0.004 |

**Supplementary Table 5.** Assessment of normality using Shapiro-Wilk normality test of difference in engagement counts between the “Pulse” video and each creator’s baseline videos’ median engagement analytics.
